# Reply to Comment on: “Impact of Depth of Invasion in Node‐Negative Oral Tongue Cancer Treated With Surgery Alone”

**DOI:** 10.1002/kjm2.70171

**Published:** 2026-01-27

**Authors:** Ming‐Hsien Tsai, Hui‐Ching Chuang, Chih‐Yen Chien

**Affiliations:** ^1^ Department of Otolaryngology Kaohsiung Chang Gung Memorial Hospital and Chang Gung University College of Medicine Kaohsiung Taiwan; ^2^ Graduate Institute of Clinical Medical Sciences College of Medicine, Chang Gung University Taoyuan Taiwan; ^3^ School of Medicine Chang Gung University College of Medicine Taoyuan Taiwan; ^4^ School of Medicine College of Medicine, National Sun Yat‐Sen University Kaohsiung Taiwan; ^5^ Doctoral Program of Clinical and Experimental Medicine National Sun Yat‐Sen University Kaohsiung Taiwan

Dear Editor,

We sincerely appreciate the opportunity to respond to the valuable comments provided regarding our study [1]. The reviewers raised several insightful points that have helped us clarify and further strengthen our work. Below, we address each major concern in detail.

First, we acknowledge that restricting the study cohort by excluding patients with nodal metastasis, adjuvant treatment, or close surgical margin may enrich the population with relatively favorable features. However, the study cohort was not composed solely of low‐risk individuals. Among the 243 patients included, 45 (18.5%) had advanced primary tumors (pT3–T4a), 31 (12.8%) exhibited perineural invasion, and 10 (4.1%) had lymphovascular invasion. These adverse pathological characteristics indicate that a meaningful proportion of higher‐risk patients remained represented. More importantly, our primary aim was not to estimate absolute survival rates but to test the hypothesis that even a 1‐mm increase in depth of invasion (DOI) confers incrementally worse oncologic outcomes. To appropriately evaluate the independent prognostic impact of DOI, it was necessary to minimize major confounding factors; therefore, we intentionally selected a more homogeneous cohort. The associations observed remained consistent with our previous findings [2].

Second, we agree that the proposed DOI‐based nomogram requires external validation before it can be adopted clinically. The absence of validation using an independent dataset is indeed a limitation of our work, and this has been explicitly stated in the manuscript. We appreciate the reviewer's suggestion, and future efforts—including prospective or multi‐institutional studies—are planned to provide more rigorous evaluation of the model. Although tumor budding and the worst pattern of invasion are increasingly recognized as important prognostic variables in oral cavity cancer [3, 4], these parameters were not consistently documented in the retrospective pathology reports available for this study. Consequently, they could not be incorporated into the current model. We fully agree that their inclusion may further enhance predictive accuracy and will consider integrating these features in future research when comprehensive pathological data become available.

Third, the concern regarding confounding by indication in interpreting the effect of elective neck dissection (END) is well taken. Patients selected for END may differ from those managed with observation in ways not fully reflected in the recorded variables. As our study was retrospective, specific factors influencing surgical decision‐making—such as clinical judgment, tumor orientation, patient preference, or surgeon practice patterns—were not systematically collected. Thus, propensity score adjustment or modeling incorporating surgical decision variables was not feasible. We agree that the observed protective effect of END should be interpreted cautiously and does not, on its own, warrant changes in clinical practice. Future prospective studies with standardized decision‐making criteria will be crucial to more definitively assess whether END mitigates the adverse prognostic influence of DOI.

We thank the reviewers once again for their thoughtful and constructive feedback, which has helped refine our work and guide future research directions.

## Conflicts of Interest

The authors declare no conflicts of interest.

## Data Availability

Data sharing not applicable to this article as no datasets were generated or analysed during the current study.
